# Methylmercury induces the expression of chemokine CCL4 *via* SRF activation in C17.2 mouse neural stem cells

**DOI:** 10.1038/s41598-019-41127-y

**Published:** 2019-03-15

**Authors:** Min-Seok Kim, Tsutomu Takahashi, Jin-Yong Lee, Takashi Toyama, Takayuki Hoshi, Shusuke Kuge, Yasuyuki Fujiwara, Akira Naganuma, Gi-Wook Hwang

**Affiliations:** 1 Laboratory of Molecular and Biochemical Toxicology, Graduate School of Pharmaceutical Sciences, Tohoku University, Aoba-ku, Sendai 980-8578 Japan; 2 Laboratory Animal Center, Daegu-Gyeongbuk Medical Innovation Foundation, Daegu, 360-4 South Korea; 3 Department of Environmental Health, School of Pharmacy, Tokyo University of Pharmacy and Life Sciences; 1432–1, Horinouchi, Hachioji, Tokyo 192–0392 Japan; 4 Laboratory of Pharmaceutical Health Sciences, School of Pharmacy, Aichi Gakuin University, 1-100 Kusumoto-cho, Chikusa-ku, Nagoya 464-8650 Japan; 5 Department of Microbiology, Faculty of Pharmaceutical Sciences, Tohoku Medical and Pharmaceutical University, Sendai, 981–8558 Japan

## Abstract

Methylmercury is an environmental pollutant that causes specific and serious damage to the central nervous system. We have previously shown that C-C motif chemokine ligand 4 (CCL4) protects cultured neural cells from methylmercury toxicity and expression of CCL4 is specifically induced in mouse brain by methylmercury. In this study, we examined the transcriptional regulatory mechanism that induces CCL4 expression by methylmercury using C17.2 mouse neural stem cells. The promoter region of the *CCL4* gene was analyzed by a reporter assay, revealing that the region up to 50 bp upstream from the transcription start site was necessary for inducing expression of CCL4 by methylmercury. Nine transcription factors that might bind to this upstream region and be involved in the induction of CCL4 expression by methylmercury were selected, and the induction of CCL4 expression by methylmercury was suppressed by the knockdown of serum response factor (SRF). In addition, the nuclear level of SRF was elevated by methylmercury, and an increase in the amount bound to the *CCL4* gene promoter was also observed. Furthermore, we examined the upstream signaling pathway involved in the induction of CCL4 expression by SRF, and confirmed that activation of p38 and ERK, which are part of the MAPK pathway, are involved. These results suggest that methylmercury induces the expression of CCL4 by activating SRF via the p38 and ERK signaling pathway. Our findings are important for elucidating the mechanism involved in the brain-specific induction of CCL4 expression by methylmercury.

## Introduction

Methylmercury is a harmful heavy metal found widely in the environment and causes central nervous disorders because this compound can cross the blood brain barrier^1,2^. Currently, the most problematic methylmercury health disorder for humans is fetal toxicity because of methylmercury exposure during pregnancy^3–5^. Methylmercury can pass through the blood placental barrier and affects the development of the immature fetal brain^6^. Methylmercury accumulates rapidly in the liver and kidneys when administered to mice, and gradually accumulates in the brain^7^. Moreover, the methylmercury concentration that accumulates in the brain is lower than that found in the liver and kidneys^7^. Nevertheless, methylmercury has brain-specific toxicity and clarifying the mechanisms associated with this toxicity is required.

We have screened for brain-specific expression of genes in mice administered with methylmercury and identified the C-C motif chemokine ligand 4 (CCL4)^8,9^. In addition, the expression of CCL4 was induced prior to methylmercury toxicity, and CCL4 had a protective effect against methylmercury toxicity in mouse neural stem cells^10^. These findings suggest that the induction of CCL4 expression by methylmercury may be a protective response to methylmercury toxicity. Therefore, elucidating the mechanism involved in the induction of CCL4 expression by methylmercury is important for understanding the brain-specific toxicity exhibited by methylmercury.

NF-κB is a major transcription factor that induces the expression of cytokines and chemokines^11,12^. It was also reported that NF-kB is involved in the induction of CCL4 expression by interleukin-1β^13^ and lipopolysaccharide^14^. However, using C17.2 mouse neural stem cells, the induction of CCL4 expression by methylmercury was slightly suppressed by knockdown of p65, which is a subunit of NF-κB^10^. This observation suggests that NF-κB is only slightly involved in the induction of CCL4 expression by methylmercury as a transcription factor, and that unknown transcription factors may be primarily responsible for regulating CCL4 expression. In this study, we aimed to elucidate the mechanisms of induction of CCL4 expression by methylmercury, and searched for transcription factors involved in this induction and investigated mechanisms related to this induction using C17.2 mouse neural stem cells.

## Results

### Identification of the promoter region related to the induction of CCL4 expression by methylmercury

**Figure 1:**
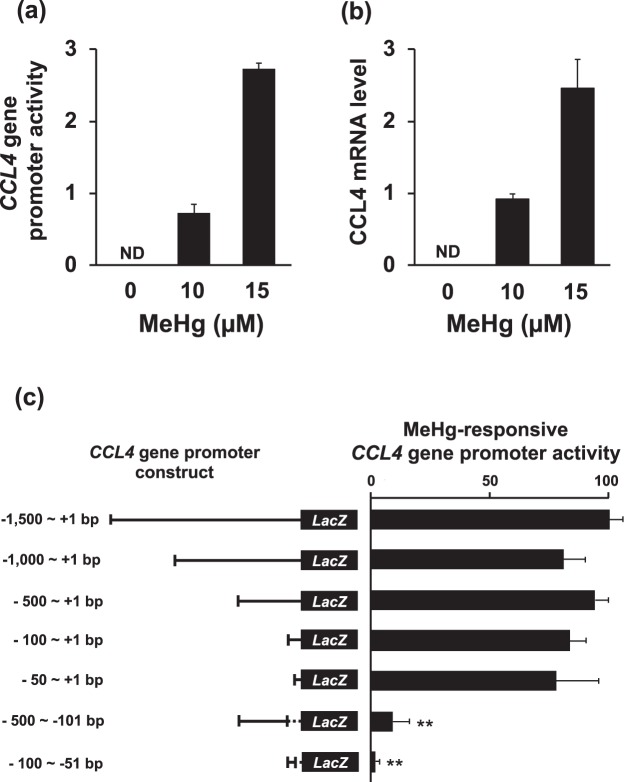
Identification of methylmercury-responsive regions in the *CCL4* gene promoter. (**a**,**c**) C17.2 cells (5 × 10^5^ cells/2 mL) were transfected with reporter plasmids containing promoter regions of the *CCL4* gene with different lengths. After incubation for 18 h, cells were treated with 10 µM methylmercuric chloride (MeHg) for 6 h. Total RNA was extracted from C17.2 cells treated with MeHg. Promoter activity was determined using quantitative real-time PCR of *LacZ* mRNA. (**b**) The mRNA level of CCL4 was measured by quantitative real-time PCR. Data are presented as mean ± S.D. ND: not detected. Statistical differences **p < 0.01 compared with 1,500 bp promoter construct group.

The promoter region that controls the induction of CCL4 expression following exposure of C17.2 cells to methylmercury was investigated by a reporter assay. The promoter activity of the *CCL4* gene was examined by sandwiching the *LacZ* as a reporter gene between the CCL4 promoter region (−1,500  to +1 bp region) and the transcription start site of the *CCL4* gene, and measuring the *LacZ* mRNA level. As shown in Fig. 1a,b, the promoter activity of the *CCL4* gene was similarly increased under the condition that the endogenous CCL4 mRNA level was increased by methylmercury. The result showed that a sequence responsive to methylmercury was contained in the 1,500 bp promoter region. To clarify the promoter region involved in the induction of CCL4 expression by methylmercury, this region was progressively shortened and the promoter activity measured. Activation of the *CCL4* gene promoter by methylmercury was observed only in a 50 bp DNA region directly upstream from the transcription start site (Fig. 1c). In addition, it was also confirmed that activation of the promoter by methylmercury was barely observed by removing the −50  to +1 bp region of the promoter while including the −51 to −100 bp region or the −101 to −500 bp region (Fig. 1c). These results indicated that the transcription start region of the *CCL4* gene to 50 bp upstream of the start site is crucial for inducing CCL4 expression by methylmercury.

### Identification of novel transcription factors related to the induction of CCL4 expression by methylmercury

**Figure 2:**
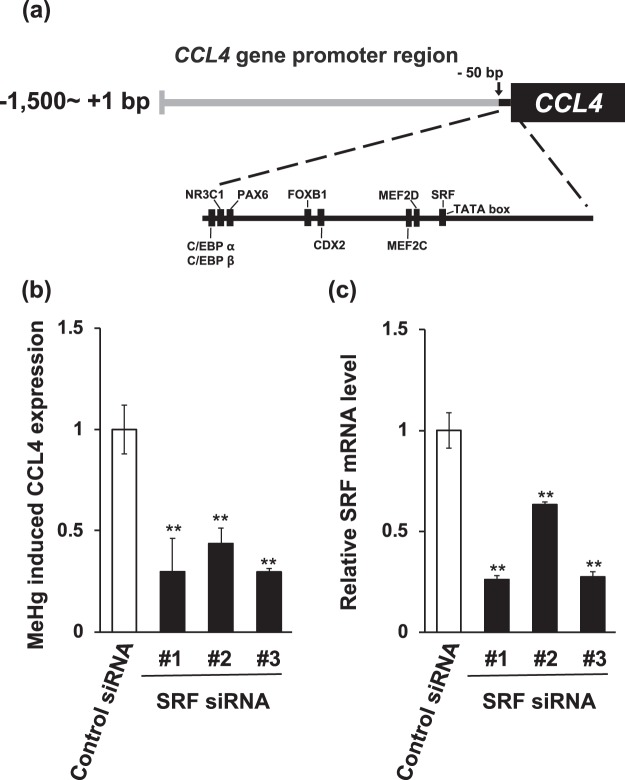
Effect of knockdown of SRF on the methylmercury-induced CCL4 expression in C17.2 cells. (**a**) There are nine transcription factor binding sites in the 50 bp promoter region of *CCL4* gene. The transcription factor that may bind to the promoter region of *CCL4* gene predicted within a dissimilarity margin less or equal than 5% by using the ALGGEN-PROMO.v8.3 online software. (**b**,**c**) C17.2 cells (5 × 10^5^ cells/2 mL) transfected with SRF siRNA were seeded onto 6-well plate. After incubation for 18 h, the transfected cells were treated with 10 µM methylmercuric chloride (MeHg) for 6 h, and mRNA level was determined by quantitative real-time PCR. Statistical differences **p < 0.01 compared with control group.

Using the ALGGEN-PROMO.v8.3 online software^15,16^, we searched for a transcription factor that may bind to the identified 50 bp promoter region of the *CCL4* gene and found nine transcription factors as candidates (Fig. 2a). Therefore, we suppressed the expression of these transcription factors to determine the possible role of these proteins in inducing CCL4 expression upon exposing the cells to methylmercury. Only knockdown of SRF suppressed elevation of the CCL4 mRNA level by methylmercury (Fig. 2b). In addition, the inhibitory efficiency of SRF expression under this condition was 45 to 75% (Fig. 2c). Therefore, SRF may be involved in the induction of CCL4 expression by methylmercury as a transcription factor.

### Methylmercury promotes the binding of SRF to the *CCL4* gene promoter

**Figure 3:**
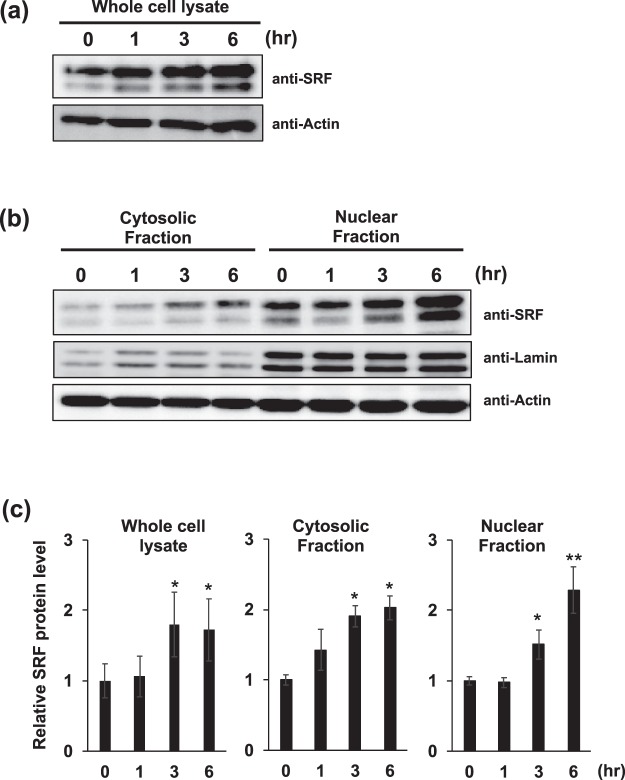
Effects of methylmercury on nuclear level of SRF in C17.2 cells. C17.2 cells (5 × 10^5^ cells/2 mL) were seeded onto each well of a 6-well plate. After incubation for 18 h, cells were treated with 10 µM methylmercuric chloride (MeHg) for the indicated times. After treatment with MeHg, the levels of SRF in whole cell lysates (**a**) and each fraction (**b**) were analyzed by immunoblotting using the indicated antibodies. Levels of actin and lamin (nuclear fractions) served as loading controls. (**c)** The ratio of SRF protein expression to actin protein expression was determined by comparing the relative intensities of protein bands from three independent experiments. Statistical differences *p < 0.05 and **p < 0.01 compared with control.

**Figure 4:**
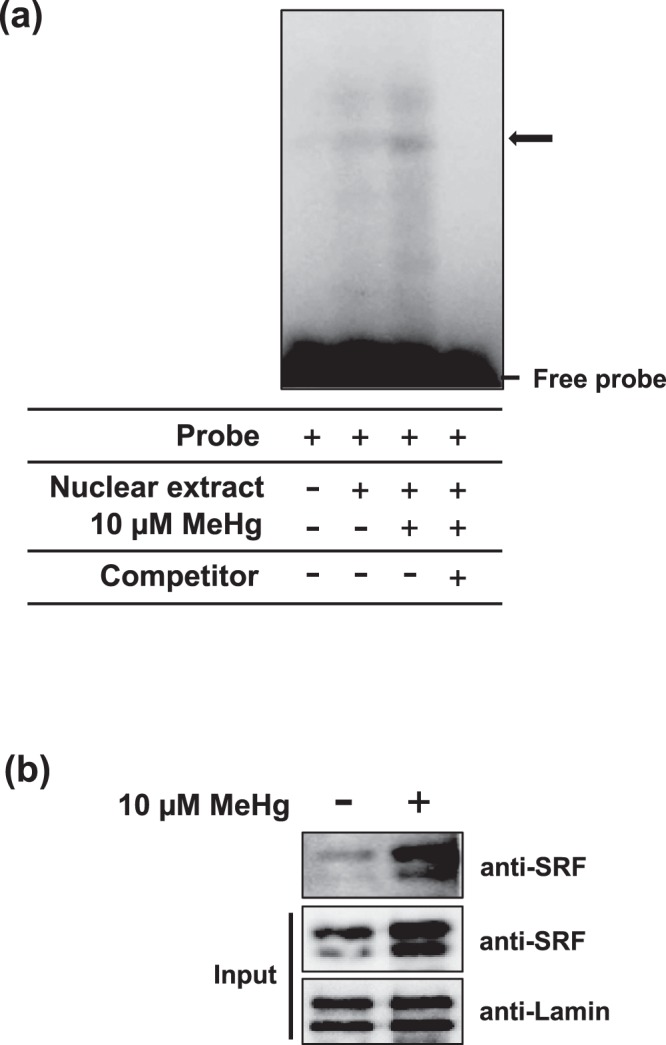
Effect of methylmercury on SRF binding to *CCL4* gene promoters. C17.2 cells (4 × 10^5^ cells/2 mL) were seeded onto each well of a 6-well plate. After incubation for 18 h, cells were treated with 10 µM methylmercuric chloride (MeHg) for 6 h and the nuclear fractions were isolated. (**a)** An electrophoretic mobility shift analysis (EMSA) was performed by incubating nuclear fractions with the ^32^P-labeled SRF consensus probe. The protein-^32^P-labeled SRF consensus probe complex is indicated by a thick arrow. The competitor is a 50-fold molar excess of the cold SRF consensus probe. (**b)** The biotinylated-SRF consensus probe was pre-incubated with streptavidin agarose beads and the nuclear extract was added. The amount of SRF bound to the biotinylated-SRF consensus probe was analyzed by immunoblotting using SRF antibody.

The effect of methylmercury on the intracellular level and distribution of SRF was investigated. Methylmercury increased the intracellular level of SRF (Fig. 3a) and also increased the level of this protein in the nucleus (Fig. 3b,c). Thus, it was shown that methylmercury may induce CCL4 expression via an increase in nuclear level of SRF. The effect of methylmercury on the binding of SRF to the *CCL4* gene promoter was investigated by EMSA, which was performed using an oligo-DNA containing the SRF binding region. The result of this assay showed that a protein with increased binding to the DNA fragment containing the binding sequence of SRF was observed in a nuclear extract obtained from methylmercury treated C17.2 cells (Figs 4a and S1). Thus, to confirm protein binding to the DNA fragment containing the binding sequence of SRF, the probe used for EMSA was labeled with biotin and the protein that bound to the probe was recovered using streptavidin agarose beads and detected by immunoblotting. Methylmercury increased the binding amount of SRF to the probe of the *CCL4* gene promoter (Fig. 4b). These findings suggested that methylmercury induces CCL4 expression via increased binding of SRF to the CCL4 gene promoter.

### Methylmercury induces CCL4 expression by activating p38 and the ERK-dependent SRF signaling pathway

**Figure 5:**
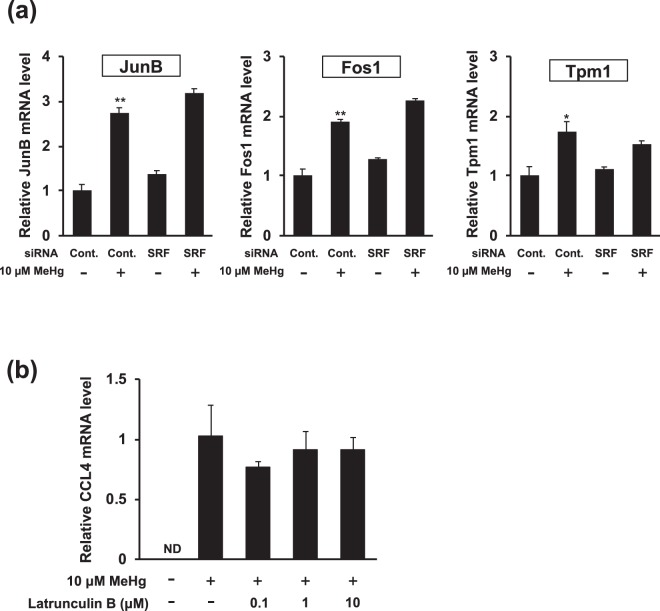
Effects of methylmercury on the mRNA levels of genes regulated by the Rho/SRF signaling pathway. (**a**) C17.2 cells (5 × 10^5^ cells/2 mL) transfected with control siRNA or SRF siRNA were seeded onto each well of a 6-well plate. After incubation for 18 h, the transfected cells were treated with 10 µM methylmercuric chloride (MeHg) for 6 h, and JunB, Fos1 and Tpm1 mRNA levels were determined by quantitative real-time PCR. Statistical differences *p < 0.05 and **p < 0.01 compared with control group. (**b**) C17.2 cells (4 × 10^5^ cells/2 mL) were seeded onto 6-well plate. After incubation for 18 h, cells were pre-treated with latrunculin B for 1 h and then incubated with 10 µM MeHg for 6 h. The CCL4 mRNA level was determined by quantitative real-time PCR. ND: not detected.

**Figure 6:**
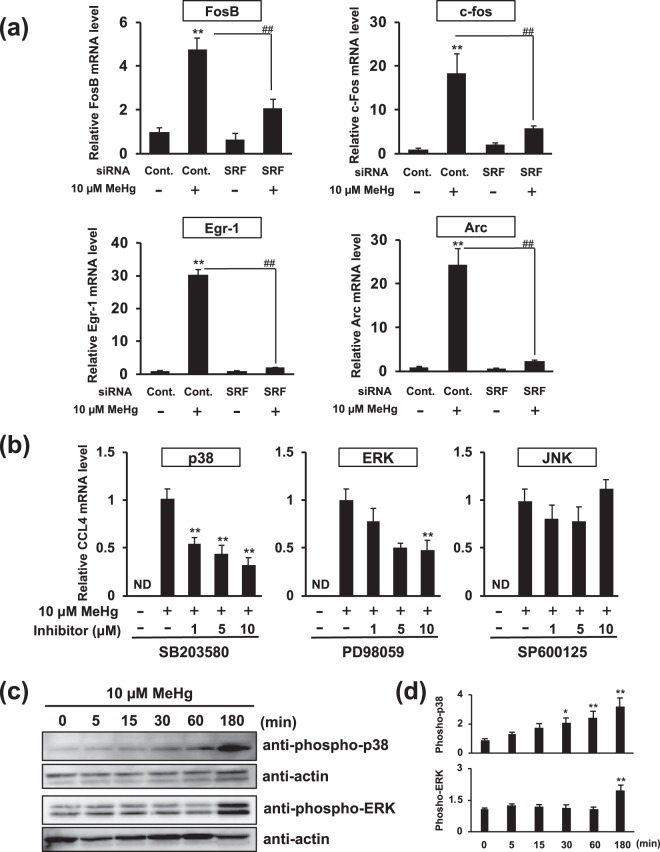
Effects of methylmercury on the mRNA levels of genes regulated by the MAPK/SRF signaling pathway. (**a**) C17.2 cells (5 × 10^5^ cells/2 mL) transfected with control siRNA or SRF siRNA were seeded onto a 6-well plate. After incubation for 18 h, the transfected cells were treated with 10 µM methylmercuric chloride (MeHg) for 6 h, and FosB, c-Fos, Egr-1 and Arc mRNA levels were determined by quantitative real-time PCR. ND: not detected. Statistical differences **p < 0.01 compared with control group. ^##^*p* < 0.01. (**b**) C17.2 cells (4 × 10^5^ cells/2 mL) were seeded onto a 6-well plate. After incubation for 18 h, cells were pre-treated with the p38 inhibitor SB203580, ERK inhibitor PD98059 or JNK inhibitor SP600125 for 1 h, and then incubated with 10 µM MeHg for 6 h. The CCL4 mRNA level was determined by quantitative real-time PCR. ND: not detected. Statistical differences **p < 0.01 compared with “absence of inhibitor, 10 µM MeHg group”. (**c**) C17.2 cells (4 × 10^5^ cells/2 mL) were seeded onto each well of a 6-well plate. After incubation for 18 h, cells were treated with 10 µM MeHg for the indicated times. After treatment with MeHg, phosphorylated p38 or ERK was detected by Western blotting. (**d**) The ratio of each phosphorylation to actin protein expression was determined by comparing the relative intensities of protein bands from three independent experiments. Statistical differences *p < 0.05 and **p < 0.01 compared with “10 µM MeHg, 0 min group”.

SRF-mediated transcription is activated by two major pathways, the Rho signaling pathway and the MAPK signaling pathway^17,18^. It has also been reported that the downstream genes transcribed by SRF differ depending on the signaling pathway involved in the activation of SRF^19^. Therefore, the signaling pathway that induces CCL4 expression via SRF activation by methylmercury was investigated. Methylmercury increased the expression level of SRF dependent genes (JunB, Fos1 amd Tpm1) via the Rho signaling pathway by about 2-fold; however, these inductions were not suppressed by SRF knockdown (Fig. 5a). The result indicates that SRF is not involved in the induction of expression of these genes by methylmercury. Moreover, CCL4 induction by methylmercury was barely affected by treatment with latrunculin B, which inhibits the Rho signaling pathway by suppressing actin depolymerization (Fig. 5b). These results indicated that Rho signaling contribute little to the induction of CCL4 expression by methylmercury. In contrast, methylmercury also significantly increased the expression level of SRF-dependent genes (FosB, c-fos, Egr-1 and Arc) via the MAPK signaling pathway and these inductions were suppressed by SRF knockdown (Fig. 6a). In addition, the effect of MAP kinase inhibitors (p38, ERK and JNK) on induction of CCL4 expression by methylmercury was investigated, and CCL4 induction was partially suppressed by inhibitors of p38 and ERK (Fig. 6b). Moreover, it was also confirmed that methylmercury enhanced phosphorylation of p38 and ERK (Fig. 6c,d). These results suggested that methylmercury induces CCL4 expression via SRF activation by phosphorylation of p38 and ERK.

### SRF is also involved in the induction of CCL3 expression by methylmercury

**Figure 7:**
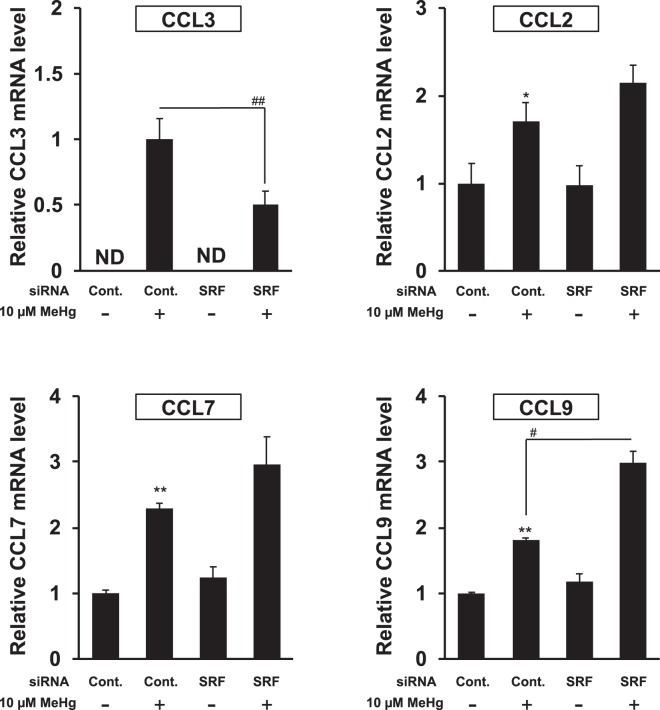
Effects of knockdown of SRF on the methylmercury-induced expression of various chemokines in C17.2 cells. C17.2 cells (5 × 10^5^ cells/2 mL) transfected with SRF siRNA were seeded onto a 6-well plate. After incubation for 18 h, the transfected cells were treated with 10 µM methylmercuric chloride (MeHg) for 6 h, and the mRNA levels of CCL2, CCL3, CCL7 and CCL9 were determined by quantitative real-time PCR. ND: not detected. Statistical differences ^**^p < 0.01 compared with control group. ^#^*p* < 0.05 and ^##^*p* < 0.01.

We reported that brain-specific expression of CCL3 in addition to CCL4 is induced in mice administered with methylmercury^9,20^. We also found that CCL2, CCL7 and CCL9 are induced not only in the mouse brain but also in the kidneys^9^. Thus, the involvement of SRF in the specificity of chemokine molecules induced by methylmercury was investigated. We examined the suppression of the expression of SRF in C17.2 cells on induction of these chemokines by methylmercury. In addition to CCL4, it was confirmed that an increase in the expression level of CCL3, in which mouse brain-specific induction was observed, by methylmercury is suppressed by SRF knockdown (Fig. 7). However, the expression levels of CCL2, CCL7 and CCL9, in which an increase was observed in both the brain and kidneys, were also increased by methylmercury, but SRF knockdown did not suppress the expression levels of these chemokines (Fig. 7). This result indicated that SRF may be specifically involved in the induction of CCL3 expression by methylmercury.

## Discussion

In this study, SRF was identified to be involved in the induction of CCL4 expression by methylmercury as a transcription factor in C17.2 mouse neural stem cells. In the cerebellum of the mouse, the amount of bound protein to the DNA probe (−28 to −19 bp of the *CCL4* gene promoter) with the SRF binding motif was also increased by methylmercury (Fig. S2). This suggests that SRF may be involved in the induction of CCL4 expression by methylmercury in mice.

SRF belongs to the MADS box family and plays a role in the muscle differentiation, migration, maintenance of cell morphology through inducing expression of cytoskeletal genes (β-actin, viculin) and immediate-early genes (c-Fos, Egr-1)^21,22^. Although induced expression of chemokines (CXCL1 and CXCL8) because of inflammatory stimulation is reduced in SRF knockout mice^23,24^, the involvement of SRF in the induction of the expression other chemokines has not been clarified. Therefore, the findings in this study that SRF is involved in inducing expression of CCL3 and CCL4 by methylmercury should aid in elucidating the novel mechanism of SRF-mediated transcription.

As shown in Fig. 3a, it was revealed that the intracellular level of SRF was elevated by methylmercury. However, we found that the expression of CCL4 was not induced only by the overexpression of SRF (data not shown). These results suggest that elevated the intracellular level of SRF may be not involved in the induction of CCL4 expression by methylmercury.

The results in Fig. 6 suggest that the activation of MAPK (p38 and ERK) is involved in the SRF-mediated transcriptional activity by methylmercury. MAPK phosphorylates the ternary complex factor (TCF), a major cofactor of SRF, and increases the transcriptional activity of SRF by promoting translocation of this factor into the nucleus^17^. Activation of SRF via the MAPK-TCF pathway in the central nervous system is involved in neuronal differentiation, dendrite formation and hippocampal formation^25–27^. Moreover, SRF has relatively low transcriptional activity but the activity of SRF is enhanced upon binding with multiple TCFs^19^. Ets-like transcription factor-1 (Elk-1), Elk-3 and Elk-4 are also major TCFs, and downstream genes whose expression is induced by SRF-binding TCFs are different^28,29^. However, suppression of each of these expressions did not affect the induction of CCL4 expression by methylmercury (data not shown). On the other hand, time course studies revealed that there are timing differences between ERK and p38 in the elevation of phosphorylation levels by methylmercury (Fig. 6c,d). Moreover, ERK and p38 are known to regulate the activity of SRF through different cofactor, respectively^19^. Based on these observations, unknown cofactors activated by ERK and/or p38 may bind SRF and participate in the induction of CCL4 expression by methylmercury. We are searching for cofactors that induce CCL4 expression by methylmercury, including proteins that bind to SRF in the presence of methylmercury. If cofactors can be identified, the detailed mechanism of induction of CCL4 expression by methylmercury via SRF activation will be revealed.

As shown in Fig. 7, SRF is specifically involved in the induction of CCL3 and CCL4 expression among chemokines that are elevated by methylmercury. Brain-specific expression of CCL3 and CCL4 is induced in mice administered with methylmercury^9^. Therefore, SRF appears to play an important role in the induction of brain-specific expression of CCL3 and CCL4 by methylmercury. This is supported by the observation that the level of SRF in the brain of mice was relatively high when compared with that of the kidney and liver (data not shown). However, because SRF is expressed in most organs, the results in this study are insufficient to explain the mechanism involved in brain-specific induction of CCL4 expression by methylmercury. As mentioned above, it is most important to identify the cofactor(s) that binds to SRF, and furthermore it is necessary to investigate the organ distribution and level in the brain of this yet to be identified cofactor(s). Nevertheless, the finding in this study that SRF is a major transcription factor involved in inducing the expression of CCL3 and CCL4 by methylmercury helps our understanding of the mechanism involved in brain-specific induction of the expression of these two chemokines. Moreover, we found that suppressing SRF expression enhances the toxicity of methylmercury toward C17.2 cells (data not shown). Our findings suggest that SRF may play an important role in the response of the central nervous system to neurotoxicity caused by methylmercury. Future efforts that elucidate the mechanism of SRF activation by methylmercury should further demonstrate the novel function of SRF on neurotoxicity.

## Materials and Methods

### Cell culture

Mouse neural stem cells (C17.2 cells) were cultured in Dulbecco’s modified Eagle medium (Nissui Pharmaceutical, Tokyo, Japan) supplemented with 10% fetal bovine serum (Biowest, Nuaillé, France), antibiotics (100 U/ml penicillin, 100 µg/ml streptomycin: Invitrogen, Carlsbad, CA, USA) and 2 mM L-glutamine (Nacalai Tesque, Kyoto, Japan) in a humidified atmosphere of 5% CO_2_ at 37 °C.

### RNA extraction and quantitative real-time RT-PCR

Total RNA was isolated from cells using the Isogen II kit (Nippon Gene, Tokyo, Japan) as described previously^30^. Synthesis of cDNA from total RNA was performed using the PrimeScript™ RT reagent kit (Takara, Shiga, Japan). Real-time PCR was performed using SYBR Premix EX Taq (Takara) and the primers shown in Table S1 as described previously^10^. The thermal condition was 95 °C for 3 min, and 40 cycles of 95 °C for 5 sec, 60 °C for 30 sec and 95 °C for 15 sec. Fold changes in mRNA levels were determined from standard curves after calibration of the assay. Each mRNA level was normalized to that of GAPDH.

### Reporter assay

The *CCL4* gene promoter region was amplified by PCR with mouse chromosomal DNA as the template and various oligonucleotides as primers (see Table S2). The PCR product were digested with MfeI and HindIII (New England Biolabs, Beverly, MA, USA) and then the fragments were ligated into the *LacZ* reporter vector pcDNA3.1-LacZ (Invitrogen), respectively. pcDNA3.1-LacZ were transfected to C17.2 cells using lipofectamine 2000 (Invitrogen). Eighteen hours after transfection, cells were treated with methylmercuric chloride for 6 h. The *CCL4* gene promoter-reporter activity was determined using quantitative real-time PCR of *LacZ* mRNA.

### siRNA transfection experiment

Double-stranded small interfering RNA (siRNA) for serum response factor (SRF, target sequence: #1, GCTCAATTTGCTATGAGTATT; #2, CGCTACACGACCTTCAGCATT; #3, CAGTGTTCCCGTCCGAGGATT) and negative control siRNA were purchased from Sigma-Aldrich (St. Louis, MO, USA). Transfection of C17.2 cells with siRNAs was carried out using the HiperFect transfection reagent (Qiagen, Germantown, MD, USA), as described previously^30^. Eighteen hours after siRNA transfection, cells were treated with methylmercuric chloride for 6 h.

### Western blotting

Methylmercury-treated C17.2 cells were washed twice with cold PBS and lysed with RIPA buffer (1 mM Tris-HCl [pH 7.4], 1% NP-40, 0.1% sodium deoxycholate, 0.1% SDS, 150 mM NaCl, 1 mM EDTA) containing cOmplete™ protease inhibitor cocktail (Roche, Indianapolis, IN, USA) and phosphatase inhibitor cocktail 2 and phosphatase inhibitor cocktail 3 (Sigma-Aldrich). Protein concentrations were measured using the DC protein assay kit (Bio-Rad Laboratories, Hercules, CA, USA). Proteins were resolved by 12.5% SDS-polyacrylamide gel electrophoresis (SDS-PAGE) and transferred to a polyvinylidene difluoride (PVDF) membrane (Millipore, Billerica, MA, USA). Western blotting was performed with primary antibodies against β-actin (Santa Cruz Biotechnology, Santa Cruz, CA, USA), SRF (Cell Signaling, Danvers, MA, USA) and horseradish peroxidase-coupled secondary antibodies (Dako A/S, Glostrup, Denmark). Immunoreactive proteins were detected by enhanced chemiluminescence using Immobilon Western chemiluminescent HRP substrate (Millipore). Chemiluminescent images were obtained using a Molecular Imager VersaDoc™ MP 5000 system (Bio-Rad Laboratories).

### Electrophoretic mobility shift assay (EMSA)

Nuclear extracts from C17.2 cells were prepared as described previously^20^. The nuclear extracts and ^32^P-labeled oligonucleotide (SRF consensus probe: GAGTCCCTATAAAGAGGGGTTC, underlining indicates SRF consensus binding site in −28 to −19 bp of the *CCL4* gene promoter) were incubated in EMSA binding buffer (10 mM Tris-HCl [pH 7.5], 4% glycerol, 1 mM MgCl_2_, 0.5 mM EDTA, 0.5 mM DTT, 50 mM NaCl) at 25 °C for 30 min. DNA-protein complexes were resolved on a 4.5% polyacrylamide gel in 0.5 × TBE (Tris-borate-EDTA) buffer. Gels were dried and subjected to autoradiography. Image analysis was performed using Typhon FLA9500 (GE Healthcare Japan, Tokyo, Japan).

### Biotinylated DNA probe-protein binding assay

The 5′-biotinylated oligonucleotide (SRF consensus probe: GAGTCCCTATAAAGAGGGGTTC) were conjugated to streptavidin agarose beads at 4 °C for 3 h. The nuclear extracts and the biotinylated oligonucleotide conjugated streptavidin agarose beads were incubated in EMSA binding buffer at 4 °C for 12 h. The beads were washed three times with Tris-buffed saline and boiled for 3 min in SDS sample buffer. Levels of SRF on binding to the biotinylated oligonucleotide were analyzed by immunoblotting using SRF antibodies.

### Statistical analysis

If not stated otherwise, the statistical significance of data was determined using one-way analysis of variance (ANOVA) with the Bonferroni post hoc test.

## Supplementary information

Supp. Tables and Figures
