# Supplementary material for: Methylmercury induces the expression of chemokine CCL4 via SRF activation in C17.2 mouse neural stem cells
Source: Sci Rep. 2019 Mar 15;9:4631. doi: 10.1038/s41598-019-41127-y (PMC6420654; doi:10.1038/s41598-019-41127-y)
Supplement: Supplementary file 1 — Supp. Tables and Figures [file 41598_2019_41127_MOESM1_ESM.pdf]

Methylmercury induces the expression of chemokine CCL4 *via* SRF activation in C17.2 mouse neural stem cells

Min-Seok Kim[1,2], Tsutomu Takahashi[1,3], Jin-Yong Lee[1,4], Takashi Toyama[1], Takayuki Hoshi[1], Shusuke Kuge[5], Yasuyuki Fujiwara[3], Akira Naganuma[1] and Gi-Wook Hwang[1,*]

[1]*Laboratory of Molecular and Biochemical Toxicology, Graduate School of Pharmaceutical Sciences, Tohoku University, Aoba-ku, Sendai 980-8578, Japan*
[2]*Laboratory Animal Center, Daegu-Gyeongbuk Medical Innovation Foundation, Daegu, 360-4, South Korea*
[3]*Department of Environmental Health, School of Pharmacy, Tokyo University of Pharmacy and Life Sciences; 1432–1, Horinouchi, Hachioji, Tokyo 192–0392, Japan*
[4]*Laboratory of Pharmaceutical Health Sciences, School of Pharmacy, Aichi Gakuin University, 1-100 Kusumoto-cho, Chikusa-ku, Nagoya 464-8650, Japan*
[5]*Department of Microbiology, Faculty of Pharmaceutical Sciences, Tohoku Medical and Pharmaceutical University, Sendai, 981–8558, Japan*

Running title: Methylmercury induces CCL4 *via* SRF activation

----

***Address for all correspondence:**
Gi-Wook Hwang, Ph.D.
Laboratory of Molecular and Biochemical Toxicology, Graduate School of Pharmaceutical Sciences, Tohoku University, Sendai 980-8578, Japan
Phone & Fax: +81-22-795-6872
E-mail: gwhwang@m.tohoku.ac.jp

Table S1. Oligonucleotide primers used for real-time PCR

**Gene**	**Sense (5'-3')**	**Antisense (5'-3')**
**SRF**	ACGACCTTCAGCAAGAGGAA	AAGCCAGTGGCACTCATTCT
**c-Fos**	CCAGTCAAGAGCATCAGCAA	AAGTAGTGCAGCCCGGAGTA
**FosB**	GAGGGAGCTGACAGATCGAC	TTCCTTAGCGGATGTTGACC
**Egr-1**	GACGAGTTATCCCAGCCAAA	GGCAGAGGAAGACGATGAAG
**Arc**	GAAGTGGTGGGAGTTCAAGC	CTCCTCAGCGTCCACATACA
**Fos1**	AGAGCTGCAGAAGCAGAAGG	CAAGTACGGGTCCTGGAGAA
**JunB**	ATGTGCACGAAAATGGAACA	CCTGACCCGAAAAGTAGCTG
**Tpm1**	GTATGAAGAGGTGGCCCGTA	CGAGTTTCAGCCTCCTTCAG
**CCL4**	CAAACCTAACCCCGAGCAACAC	GGTCTCATAGTAATCCATCACAAAGC
**LacZ**	ACTATCCCGACCGCCTTACT	TAGCGGCTGATGTTGAACTG
**GAPDH**	AACTTTGGCATTGTGGAAGG	ACACATTGGGGGTAGGAACA

Table S2. Oligonucleotide primers used for *CCL4* gene promoter-reporter genes constructions

***CCL4* gene promoter**	**Sense (5'-3')**	**Antisense (5'-3')**
**CCL4 -1,500 ~ +1 bp**	CGGCAATTGCTTTGACCTTACTTGGAACT	CGCAAGCTTTGATCTGAGTTGGGAACCCC
**CCL4 -1,000 ~ +1 bp**	CGGCAATTGACTGCTCTGTCTTTTCCAGC	CGCAAGCTTTGATCTGAGTTGGGAACCCC
**CCL4 -500 ~ +1 bp**	CGGCAATTGATGATGCTGATTGAATGGTA	CGCAAGCTTTGATCTGAGTTGGGAACCCC
**CCL4 -100 ~ +1 bp**	CGGCAATTGGACATCATCTTTACTCATGA	CGCAAGCTTTGATCTGAGTTGGGAACCCC
**CCL4 -50 ~ +1 bp**	CGGCAATTGCTTGTCTTAGGCCCCAGAGT	CGCAAGCTTTGATCTGAGTTGGGAACCCC
**CCL4 -500 ~ +101 bp**	CGGCAATTGATGATGCTGATTGAATGGTA	CGCAAGCTTTCAGAAGAGTTGGTTTAGCC
**CCL4 -100 ~ +51 bp**	CGGCAATTGGACATCATCTTTACTCATGA	CGCAAGCTTGGTTGGGGTTGCAGGAAGGA

Underlining indicates MfeI restriction sites and double underlining indicates HindIII restriction sites.

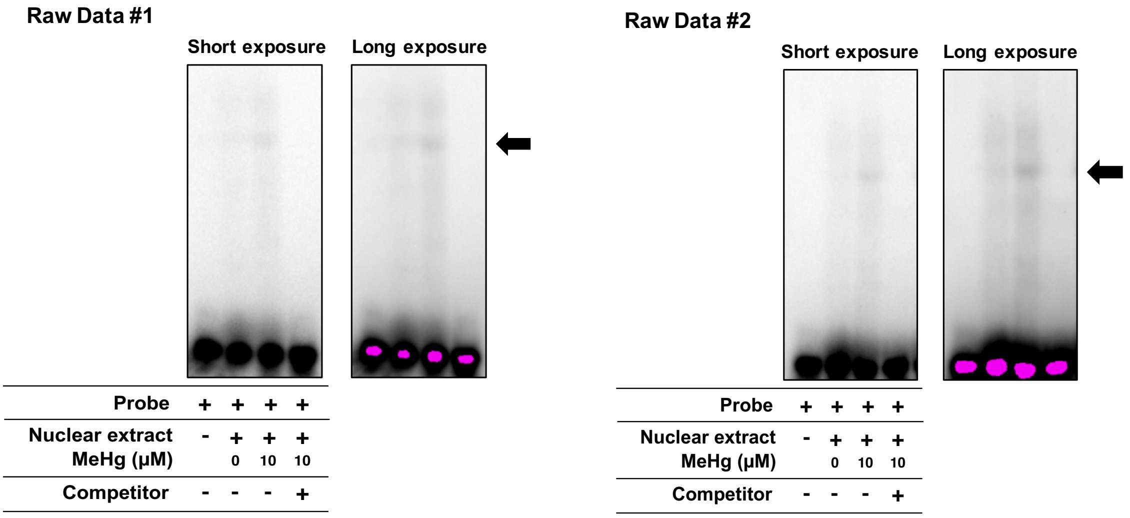

Figure S1. Effect of methylmercury on SRF binding to *CCL4* gene promoters. C17.2 cells (4×10^5 cells/2 mL) were seeded onto each well of a 6-well plate. After incubation for 18 h, cells were treated with 10 µM methylmercuric chloride (MeHg) for 6 h and the nuclear fractions were isolated. An electrophoretic mobility shift analysis (EMSA) was performed by incubating nuclear fractions with the ^{32}P-labeled SRF consensus probe. The protein-^{32}P-labeled SRF consensus probe complex is indicated by a thick arrow. The competitor is a 50-fold molar excess of the cold SRF consensus probe.

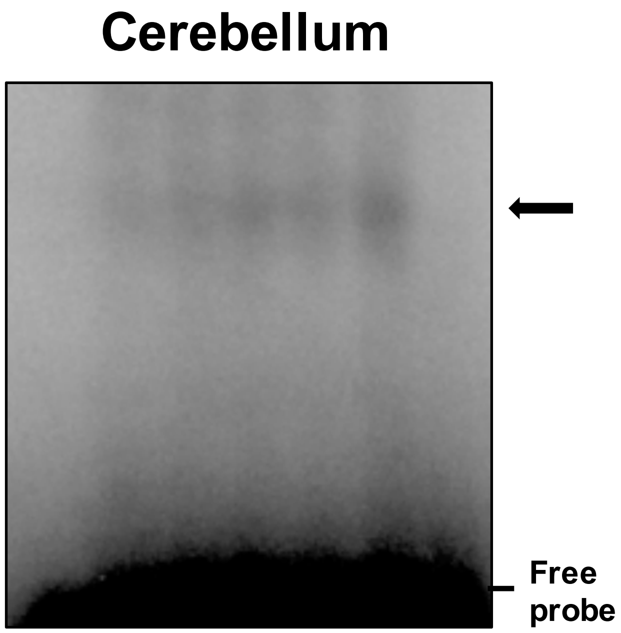

**Probe**	+	+	+	+	+	+	+
**Nuclear extract**	-	+	+	+	+	+	+
**MeHg treatment (days)**		0	1	3	5	7	7
**Competitor**	-	-	-	-	-	-	+

Figure S2. Effect of methylmercury on SRF binding to the *CCL4* gene promoter in the cerebellum of mice. C57BL/6 mice were injected subcutaneously with methylmercuric chloride (MeHg; 25 mg/kg weight). Nuclear fractions were isolated from cerebellum dissected 1, 3, 5, or 7 days after the injection. EMSA was performed by incubating nuclear fractions with the ^{32}P-labeled DNA probe (–28 to –19 bp of the *CCL4* gene promoter). The protein-^{32}P-labeled DNA probe complex is indicated by a thick arrow. The competitor is a 50-fold molar excess of the cold DNA probe.
